# Refractory Unilateral Diffuse Scleritis With Negative Autoimmune Workup Successfully Treated With Sub-tenon Triamcinolone Injection

**DOI:** 10.7759/cureus.104541

**Published:** 2026-03-02

**Authors:** Narayana Swamy, Saraswathi Saiprasad, Kristi Kway, David Garate

**Affiliations:** 1 Rheumatology, Baylor Scott and White Health, Fort Worth, USA; 2 Endocrinology, Diabetes and Metabolism, Baylor Scott and White Health, Fort Worth, USA; 3 Internal Medicine, Baylor Scott and White All Saints Medical Center, Fort Worth, USA; 4 Internal Medicine, School of Medicine at Texas Christian University, Fort Worth, USA; 5 Internal Medicine, Baylor Scott and White Health, Fort Worth, USA

**Keywords:** idiopathic scleritis, intrascleral triamcinolone, refractory ocular inflammation, scleritis, unilateral scleritis

## Abstract

Noninfectious anterior scleritis is a painful, potentially vision-threatening inflammatory disorder that may become steroid dependent despite negative systemic evaluation, posing significant management challenges. We report a man in his early sixties with an approximately eight-year history of recurrent unilateral diffuse anterior scleritis who was referred for rheumatology evaluation after infectious etiologies were excluded by ophthalmology. His disease repeatedly improved with high-dose systemic corticosteroids but relapsed during tapering, resulting in prolonged steroid dependence.

Extensive systemic evaluation, including autoimmune and vasculitis testing with antinuclear antibody, extractable nuclear antigen panel, complement levels, and antineutrophil cytoplasmic antibody testing with proteinase-3 and myeloperoxidase antibodies, was repeatedly unremarkable. Steroid-sparing therapy with methotrexate and subsequent escalation to rituximab failed to achieve a durable remission. Ophthalmology-directed intrascleral triamcinolone resulted in sustained resolution and allowed successful discontinuation of systemic corticosteroids, supporting localized corticosteroid therapy as an effective steroid-sparing option in selected cases.

## Introduction

Scleritis is a rare, sight-threatening inflammation of the sclera that causes severe ocular pain, redness, and potential vision loss. It is broadly classified into anterior and posterior forms, with anterior scleritis further subdivided into diffuse, nodular, and necrotizing subtypes; diffuse anterior scleritis is the most common subtype seen in clinical practice and often necessitates systemic therapy for adequate disease control [1]. 

Up to half of patients with scleritis have an associated systemic autoimmune disease, most commonly rheumatoid arthritis or antineutrophil cytoplasmic antibody (ANCA)-associated vasculitis [2,3]. Because ocular inflammation may precede systemic manifestations, comprehensive rheumatologic evaluation and longitudinal surveillance are recommended even when initial testing is negative [4]. 

Management of scleritis typically involves systemic nonsteroidal anti-inflammatory drugs (NSAIDs) or corticosteroids, followed by steroid-sparing immunomodulatory therapy (IMT) in recurrent or severe cases. Methotrexate is frequently used as first-line IMT, while biologic agents such as rituximab may be considered for refractory disease [5,6]. Despite these therapies, some patients remain steroid-dependent or experience frequent relapses, leading to cumulative toxicity.

Localized corticosteroid therapy, including subconjunctival or sub-tenon triamcinolone injections, has emerged as a potential adjunctive treatment for selected cases of noninfectious, non-necrotizing anterior scleritis. Although concerns regarding scleral thinning and necrosis exist, multiple studies have demonstrated favorable efficacy and safety profiles when infection and necrotizing disease are excluded [7].

We describe a case of long-standing unilateral diffuse scleritis with an unremarkable autoimmune workup that proved refractory to systemic immunosuppression but achieved durable remission following sub-tenon triamcinolone therapy.

## Case presentation

A Caucasian man in his early sixties was referred to rheumatology for evaluation of chronic, recurrent, unilateral noninfectious left-sided scleritis. He reported an approximately eight-year history of episodic severe ocular pain and redness involving the left eye. Multiple prior flares had been treated with systemic corticosteroids, with consistent clinical improvement during high-dose therapy but relapse during tapering.

**Figure 1:**
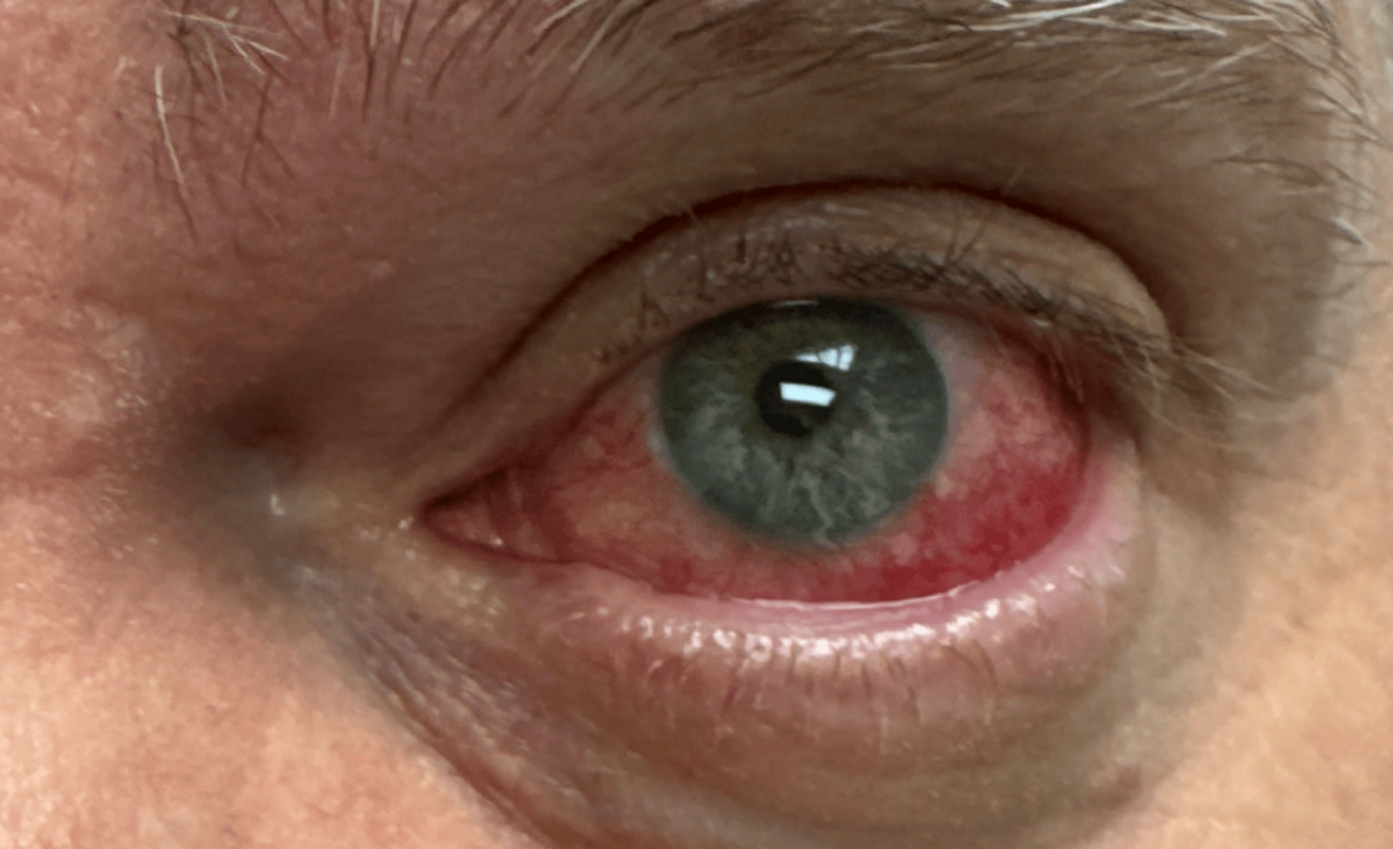
Diffuse left anterior scleritis at initial presentation. Clinical photograph of the left eye at initial presentation demonstrating diffuse anterior scleritis, characterized by marked sectoral scleral injection with deep violaceous discoloration consistent with active inflammation.

At the time of rheumatology evaluation, ophthalmologic examination had demonstrated findings consistent with diffuse anterior scleritis of the left eye (Figure 1). There was no evidence of necrotizing disease or posterior involvement. The patient also reported a history of recurrent sinus symptoms but denied systemic constitutional symptoms, inflammatory arthritis, oral ulcers, skin rashes, hematuria, pulmonary symptoms, or neurologic manifestations.

Given the chronicity and steroid dependence of his ocular disease, a comprehensive evaluation for an underlying systemic inflammatory or systemic vasculitic disorder was undertaken. Laboratory testing included antinuclear antibody (ANA) testing with reflex titers, extractable nuclear antigen (ENA) panel, complement levels, and antineutrophil cytoplasmic antibody (ANCA) testing with proteinase-3 (PR3) and myeloperoxidase (MPO) antibodies. These studies were repeatedly unremarkable. Infectious screening prior to initiation of immunosuppressive therapy included negative QuantiFERON-TB Gold testing, unremarkable hepatitis B and hepatitis C serologies, and negative COVID-19 serology. Additional infectious evaluation performed by ophthalmology prior to rheumatology referral was also reported as negative. No systemic autoimmune or infectious etiology was identified. Autoimmune serologic laboratory studies are summarized in Table 1.

**Table 1:** Autoimmune serologic evaluation.

Test	Result	Reference Range
Antinuclear antibody (ANA)	Negative	Negative
ANA screen, indirect immunofluorescence assay (IFA)	Negative	Negative
Antineutrophil cytoplasmic antibody (ANCA) screen (1:20 dilution)	Negative	Negative
Myeloperoxidase (MPO) antibody	Negative	Negative
Proteinase 3 (PR3) antibody	Negative	Negative
Double-stranded DNA (dsDNA) antibody	<1 IU/mL	≤ 4 IU/mL negative; 5-9 IU/mL indeterminate; ≥ 10 IU/mL positive
Anti-SSA (Ro) antibody	<1.0 (negative)	<1.0
Anti-SSB (La) antibody	<1.0 (negative)	<1.0
Smith/RNP (ENA) antibody	<1.0 (negative)	<1.0
Smith (Sm) antibody	<1.0 (negative)	<1.0
Scleroderma (Scl-70) antibody	<1.0 (negative)	<1.0

**Figure 2:**
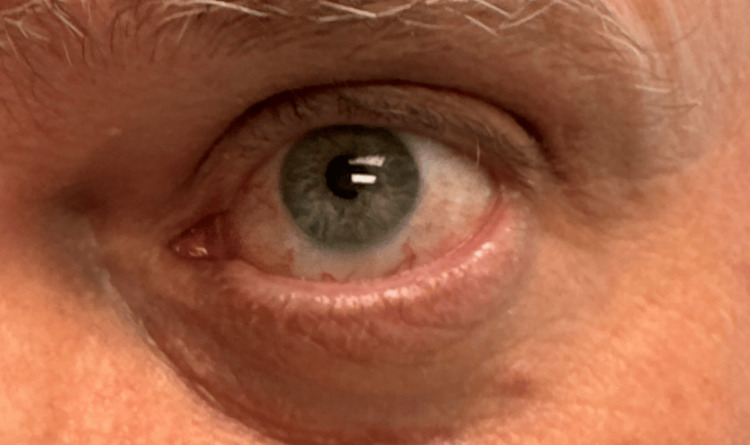
Resolved anterior scleritis in left eye. Left eye demonstrating clinical resolution of diffuse anterior scleritis, with marked reduction of deep scleral injection and restoration of normal scleral coloration following treatment.

The patient was treated with oral prednisone 60 mg daily for four weeks, resulting in the resolution of active scleritis (Figure 2).

**Figure 3:**
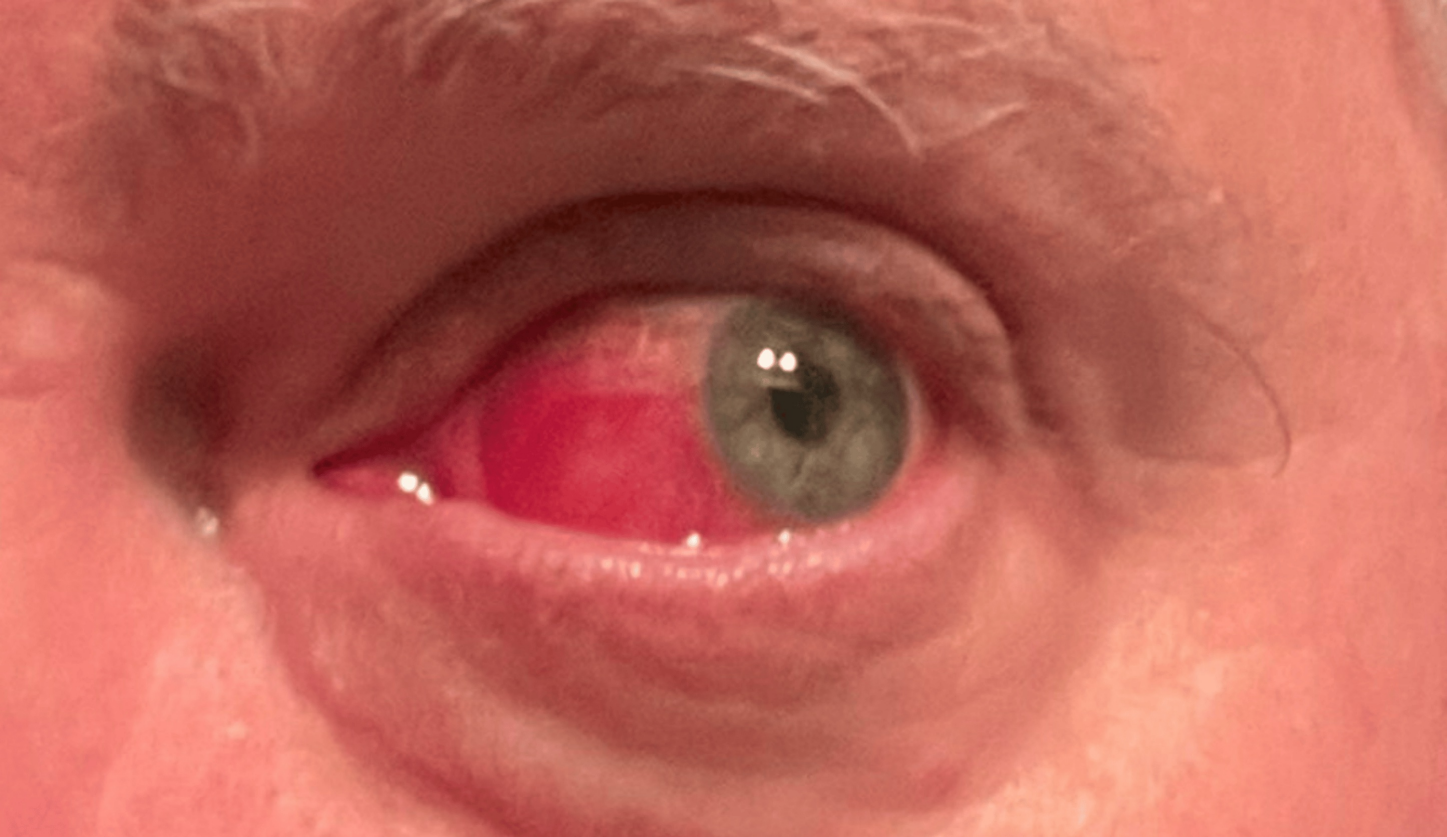
Recurrent diffuse anterior scleritis, left eye, during corticosteroid taper Recurrence of diffuse anterior scleritis in the left eye following taper of oral prednisone below 20 mg daily, demonstrating marked sectoral scleral injection and active inflammation.

The prednisone dose was then tapered by 10 mg per week to 40 mg daily, followed by a slower taper of 5 mg per week thereafter. However, when the prednisone dose was reduced to below 20 mg daily, recurrent scleritis developed (Figure 3). Multiple tapering attempts over time produced a similar relapse pattern, establishing steroid dependence.

To minimize long-term corticosteroid exposure, oral methotrexate was initiated as a steroid-sparing agent and escalated to a maximum dose of 20 mg once weekly with folic acid supplementation and appropriate laboratory monitoring. Despite this escalation, disease recurrence continued during prednisone tapering, and durable remission was not achieved.

During one severe flare while under rheumatology care, the patient received an intramuscular depot methylprednisolone (Depo-Medrol) injection, which resulted in temporary symptomatic improvement. Intravenous pulse glucocorticoid therapy was considered but not pursued, given the pattern of steroid responsiveness with rapid relapse during tapering, concerns regarding cumulative corticosteroid toxicity, and absence of necrotizing or posterior scleritis requiring emergent pulse therapy.

Given persistent disease activity and concern for progressive scleral inflammation, biologic therapy with intravenous rituximab was initiated using a rheumatologic dosing protocol of 1 g administered every two weeks for two doses. Following rituximab infusion and concurrent prednisone tapering, clinical remission was achieved for approximately three months. However, unilateral recurrence subsequently occurred, again necessitating systemic corticosteroid escalation.

During the course of prolonged corticosteroid therapy, the patient developed a lower-extremity deep venous thrombosis complicated by bilateral pulmonary emboli. Further hematologic evaluation revealed a heterozygous factor V Leiden mutation, and long-term anticoagulation was initiated under hematology supervision.

**Figure 4:**
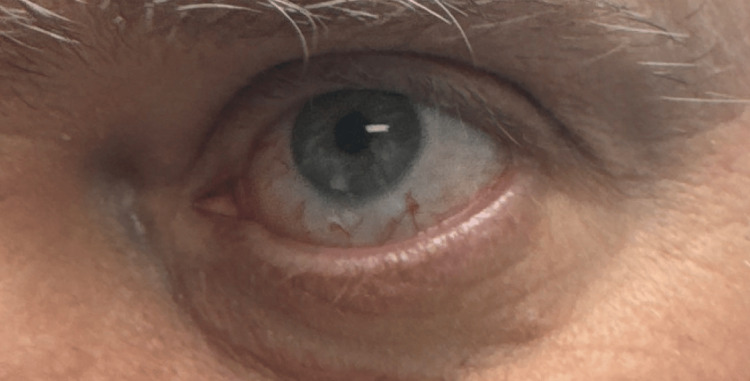
Left eye following sub-tenon triamcinolone injection. Clinical photograph of the left eye following administration of sub-tenon triamcinolone 40 mg after subconjunctival 2% lidocaine anesthesia. This targeted local corticosteroid delivery was selected to achieve deeper scleral penetration in the setting of chronic, localized anterior scleritis.

Given refractory unilateral disease despite systemic immunosuppression and the risks associated with continued corticosteroid exposure, the patient was referred to a uveitis specialist for further management. The ophthalmology team elected to administer sub-tenon triamcinolone 40 mg (Kenalog) injection after an injection of subconjunctival 2% lidocaine anesthesia to provide targeted local anti-inflammatory therapy. This route was selected to allow deeper scleral delivery in the setting of chronic localized inflammation. Following the procedure, the scleritis resolved, and systemic corticosteroids were successfully tapered and discontinued. At six months of follow-up, the patient remained in sustained remission without recurrence, and repeat autoimmune serologic testing remained unremarkable (Figure 4).

An ultra-minimal case overview summarizing the clinical course, diagnostic evaluation, treatment escalation, and outcome is provided in Table 2.

**Table 2:** Ultra-minimal case overview. Ultra-compact summary of the clinical course, diagnostic evaluation, treatment escalation, and outcome of chronic unilateral noninfectious anterior scleritis.

Item	Details
Patient	Male, early 60s
Diagnosis	Chronic unilateral noninfectious anterior scleritis
Disease course	Steroid-responsive, steroid-dependent
Systemic evaluation	Autoimmune and vasculitic workup negative
Treatments	Systemic corticosteroids → methotrexate → rituximab
Definitive therapy	sub-tenon triamcinolone
Outcome	Sustained remission at 6 months

## Discussion

This case illustrates several important considerations in the management of chronic, recurrent anterior scleritis, particularly when inflammation remains unilateral and localized in the absence of an identifiable systemic autoimmune disorder. Although up to 50% of patients with scleritis have an associated systemic inflammatory disease, a substantial subset remains idiopathic despite extensive evaluation [1,2]. In such patients, prolonged disease courses characterized by corticosteroid responsiveness with relapse during tapering may create both diagnostic uncertainty and therapeutic challenges for rheumatologists and ophthalmologists.

Systemic immunosuppression remains the cornerstone of therapy for recurrent or severe scleritis, especially when systemic disease is present or strongly suspected [5]. Methotrexate is frequently employed as a first-line steroid-sparing agent, with escalation to biologic therapies such as rituximab in refractory cases [5,6]. Published cohorts suggest variable remission rates with conventional immunosuppressive agents, and a subset of patients continue to experience relapsing disease despite escalation to biologics [6]. In this case, despite appropriately dosed systemic corticosteroids and subsequent immunomodulatory therapy, inflammation persisted and recurred during steroid tapering, without development of systemic autoimmune features during follow-up.

An important teaching point highlighted by this case is the need to reconsider treatment strategy when clinical features do not align with expected disease behavior. Persistent unilateral involvement, repeatedly negative autoimmune and vasculitis evaluations, and failure of systemic steroid-sparing agents should prompt reassessment of whether ongoing systemic immunosuppression remains the most appropriate strategy [2-4]. Continued reliance on systemic corticosteroids in this context exposes patients to cumulative toxicity, including metabolic, cardiovascular, and infectious risks, without reliably achieving durable remission [5].

Objective assessment of disease activity is critical in guiding management decisions. While standardized grading scales were not prospectively applied at every visit, clinical documentation consistently described deep scleral injection, localized inflammation, and response to therapy, supported by serial clinical photography. Imaging and laboratory evaluation were performed to exclude infectious and systemic inflammatory etiologies. The persistent localization of inflammation without necrosis or systemic correlation further supported consideration of a targeted local approach.

Localized corticosteroid therapy has emerged as an effective adjunctive option for carefully selected patients with noninfectious, non-necrotizing anterior scleritis [7-9]. Subconjunctival and sub-tenon triamcinolone injections allow targeted delivery of anti-inflammatory medication directly to the site of disease, often resulting in rapid symptom control and reduction in systemic corticosteroid requirements [7,8]. Historical concerns regarding scleral thinning or necrosis limited early adoption; however, multicenter and long-term studies have demonstrated favorable safety and efficacy profiles when infection and necrotizing disease are rigorously excluded [9]. Reported remission rates following local corticosteroid injection are high in appropriately selected patients, with relatively low rates of serious complications [7-9].

In this patient, ophthalmology-directed sub-tenon triamcinolone resulted in sustained remission after years of steroid dependence and failure of systemic immunosuppression. Systemic corticosteroids were successfully discontinued without recurrence during follow-up. This outcome underscores the importance of individualized treatment strategies and highlights that persistent localized inflammation may respond more effectively to targeted therapy than to systemic immunosuppression alone.

This case also emphasizes the value of early multidisciplinary collaboration. Close coordination between rheumatology and ophthalmology facilitated reassessment of the treatment paradigm and allowed safe implementation of localized therapy. Such collaboration is especially important in cases where disease behavior deviates from typical systemic autoimmune patterns.

Several limitations should be acknowledged. This is a single-patient report with limited long-term follow-up, which restricts generalizability. Standardized clinical grading scores and prospective imaging metrics were not uniformly recorded throughout the disease course. Additionally, spontaneous fluctuation in disease activity cannot be entirely excluded. Therefore, while this case supports consideration of localized corticosteroid therapy in carefully selected patients with refractory, localized anterior scleritis and negative systemic evaluation, broader conclusions require confirmation in larger, prospective studies.

Ultimately, optimal management of complex scleritis requires individualized assessment, careful exclusion of infectious and necrotizing etiologies, and flexibility to deviate from conventional systemic treatment algorithms when clinical circumstances warrant [10,11]. Early consideration of targeted local therapy may reduce cumulative systemic corticosteroid exposure and improve patient outcomes in select cases.

## Conclusions

Refractory unilateral diffuse anterior scleritis may persist for years despite extensive negative autoimmune and vasculitis evaluation and may remain dependent on systemic corticosteroids. This case demonstrates that ophthalmology-directed sub-tenon triamcinolone injection can achieve effective and sustained disease control in a carefully selected patient with noninfectious anterior scleritis when systemic immunosuppression failed to provide durable remission.

In patients with persistently localized disease and unremarkable systemic evaluation, prolonged reliance on systemic corticosteroids should prompt reconsideration of treatment strategy. Localized corticosteroid therapy may be considered as a steroid-sparing adjunct in selected cases, particularly when conventional systemic therapies are ineffective and infectious and necrotizing etiologies have been excluded. Given the single-patient design and limited duration of follow-up, broader generalization is not warranted. Nevertheless, this case highlights the importance of individualized management, early multidisciplinary collaboration between rheumatology and ophthalmology, and continued longitudinal surveillance to monitor for evolving systemic disease.
